# Supplementary material for: Structural insights into transcriptional regulation by the helicase RECQL5
Source: Nat Struct Mol Biol. 2025 Jul 7;32(9):1721–30. doi: 10.1038/s41594-025-01611-8 (PMC12262184; doi:10.1038/s41594-025-01611-8)
Supplement: Supplementary file 1 — Supplementary Fig. 1. [file 41594_2025_1611_MOESM1_ESM.pdf]

nature structural & molecular biology

Supplementary information https://doi.org/10.1038/s41594-025-01611-8

Structural insights into transcriptional regulation by the helicase RECQL5

In the format provided by the authors and unedited

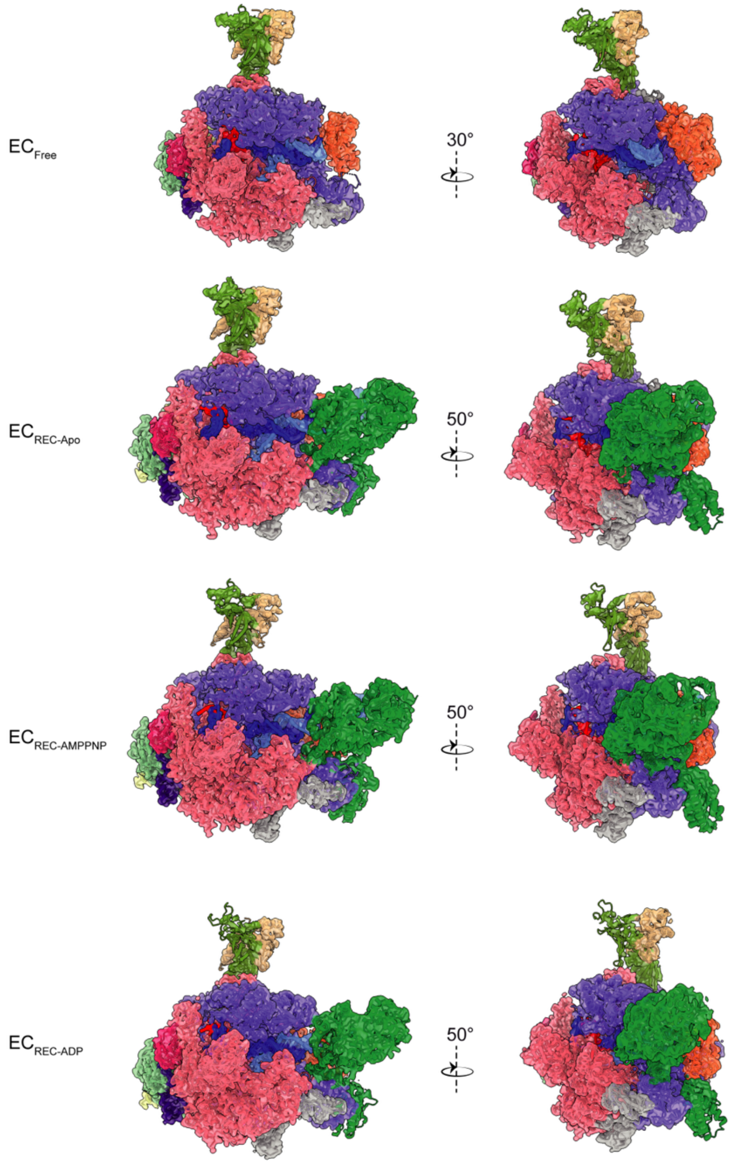

Supplementary Fig. 1 | Additional views showing model fit to map. Cryo-EM maps (shown in transparency) with fitted atomic models (shown in ribbon representation). Each map/model pair is shown in two distinct views.
