# Supplementary material for: Structural insights into transcriptional regulation by the helicase RECQL5
Source: Nat Struct Mol Biol. 2025 Jul 7;32(9):1721–30. doi: 10.1038/s41594-025-01611-8 (PMC12262184; doi:10.1038/s41594-025-01611-8)
Supplement: Supplementary file 2 — Reporting Summary [file 41594_2025_1611_MOESM2_ESM.pdf]

nature portfolio

Corresponding author(s): Eva Nogales

Last updated by author(s): May 19, 2025

Reporting Summary

Nature Portfolio wishes to improve the reproducibility of the work that we publish. This form provides structure for consistency and transparency in reporting. For further information on Nature Portfolio policies, see our Editorial Policies and the Editorial Policy Checklist.

Statistics

For all statistical analyses, confirm that the following items are present in the figure legend, table legend, main text, or Methods section.

n/a	Confirmed	
☐	☒	The exact sample size (*n*) for each experimental group/condition, given as a discrete number and unit of measurement
☒	☐	A statement on whether measurements were taken from distinct samples or whether the same sample was measured repeatedly
☒	☐	The statistical test(s) used AND whether they are one- or two-sided<br>*Only common tests should be described solely by name; describe more complex techniques in the Methods section.*
☒	☐	A description of all covariates tested
☒	☐	A description of any assumptions or corrections, such as tests of normality and adjustment for multiple comparisons
☒	☐	A full description of the statistical parameters including central tendency (e.g. means) or other basic estimates (e.g. regression coefficient) AND variation (e.g. standard deviation) or associated estimates of uncertainty (e.g. confidence intervals)
☒	☐	For null hypothesis testing, the test statistic (e.g. *F*, *t*, *r*) with confidence intervals, effect sizes, degrees of freedom and *P* value noted<br>*Give P values as exact values whenever suitable.*
☒	☐	For Bayesian analysis, information on the choice of priors and Markov chain Monte Carlo settings
☒	☐	For hierarchical and complex designs, identification of the appropriate level for tests and full reporting of outcomes
☒	☐	Estimates of effect sizes (e.g. Cohen's *d*, Pearson's *r*), indicating how they were calculated

Our web collection on statistics for biologists contains articles on many of the points above.

Software and code

Policy information about availability of computer code

Data collection	SerialEM (v4.1.0beta) was used for cryo-EM data collection. Typhoon FLA 9500 (v1.1) was used to scan gels for the RNA extension assay.
Data analysis	cryoSPARC (v4.5.3) and RELION (v5) were used for cryo-EM data processing. Coot (v0.9.8.7) and Phenix (v1.20) were used for model building and refinement. AlphaFold (v3) was used to generate an initial model for part of RECQL5. ChimeraX (v1.8) was used to visualize and interpret structures. Python (v3.9.6) was used to visualize FSC curve plots from RELION and Phenix output files. Adobe Photoshop 2023 was used to process RNA extension assay raw images (only adjustments to tonal range were performed, and all adjustments were applied to the whole image). Clustal Omega (v1.2.4) (through EMBL-EBI, https://www.ebi.ac.uk/jdispatcher/msa/clustalo) and Jalview (v2.11.4.1) were used to align sequences and visualize evolutionary conservation, respectively. Adobe Illustrator 2023 was used to create final figures.

For manuscripts utilizing custom algorithms or software that are central to the research but not yet described in published literature, software must be made available to editors and reviewers. We strongly encourage code deposition in a community repository (e.g. GitHub). See the Nature Portfolio guidelines for submitting code & software for further information.

Data

Policy information about availability of data

All manuscripts must include a data availability statement. This statement should provide the following information, where applicable:

- Accession codes, unique identifiers, or web links for publicly available datasets
- A description of any restrictions on data availability
- For clinical datasets or third party data, please ensure that the statement adheres to our policy

All data pertaining to this paper are provided within the paper or accessible from public repositories. The cryo-EM density maps and their respective atomic coordinate files have been deposited to the Electron Microscopy Data Bank (EMDB) and Protein Data Bank (PDB) under the following accession codes: EMD-48071 and PDB 9EHZ (ECFree), EMD-48073 and PDB 9EI1 (ECREC-Apo), EMD- 48074 and PDB 9EI2 (ECREC-Apo (IRI Focused)), EMD- 48075 and PDB 9EI3 (ECREC-AMPPNP), and EMD-48076 and PDB 9EI4 (ECREC-ADP). Raw cryo-EM movies have been deposited to the Electron Microscopy Public Image Archive (EMPIAR) under the following accession codes: EMPIAR-12711 (dataset for ECFree, ECREC-Apo, and ECREC-Apo (IRI Focused)), EMPIAR-12721 (dataset for ECREC-AMPPNP), and EMPIAR-12722 (dataset for ECREC-ADP). In addition to the structures reported in this work, the following publicly available structures were used: PDB accession codes 2VUM, 1I6H, 6EXV, 5FLM, 5LB8, 8JH2, and 7NW0. Source data are provided with the manuscript.

Research involving human participants, their data, or biological material

Policy information about studies with human participants or human data. See also policy information about sex, gender (identity/presentation), and sexual orientation and race, ethnicity and racism.

Reporting on sex and gender	N/A
Reporting on race, ethnicity, or other socially relevant groupings	N/A
Population characteristics	N/A
Recruitment	N/A
Ethics oversight	N/A

Note that full information on the approval of the study protocol must also be provided in the manuscript.

Field-specific reporting

Please select the one below that is the best fit for your research. If you are not sure, read the appropriate sections before making your selection.

☒ Life sciences ☐ Behavioural & social sciences ☐ Ecological, evolutionary & environmental sciences

For a reference copy of the document with all sections, see nature.com/documents/nr-reporting-summary-flat.pdf

Life sciences study design

All studies must disclose on these points even when the disclosure is negative.

Sample size	Cryo-EM datasets were collected such that the number of particles in final classes were sufficient to yield high-resolution structures, based on empirical findings and prior experience. For the RNA extension assay, we performed this in singlicate but replicated the results in 3 independent experiments, which is standard in the field (for example, see Su & Vos, Mol. Cell 84, 1243, 2024). Other miscellaneous gels were run once, which is standard in the field and acceptable as they are only for sample validation purposes.
Data exclusions	Micrographs and particles were discarded following criteria that are standard to the field (e.g., micrographs with poor CTF fit resolution and particles corresponding to graphene oxide edges and water ice were discarded). We also performed detailed 3D classification to select particles belonging to states of interest. 3D classification is standard in the field. All details are provided in the Methods and data processing figures (Extended Data Figs. 1, 3, 5, 8, and 9).
Replication	Cryo-EM structures were not replicated, which is standard. RNA extension assay was performed 3 times with consistent results, which is standard in the field.
Randomization	Not applicable to our study. None of the experiments conducted involved dividing samples/organisms/participants into experimental groups. For purposes of cryo-EM validation, particles were randomly divided into two half-sets during image processing following established standards in the field ("gold-standard" FSC procedure). Randomization was performed using cryoSPARC, a standard program used in the field.
Blinding	Investigators were not blinded, which is standard for the cryo-EM field. Blinding is not routine in structural/biochemical studies, in part due to its impracticality in the context of structural/biochemical procedures and also since in many cases it is important to know sample identity when preparing cryo-EM samples, collecting cryo-EM data, and processing cryo-EM data.

Reporting for specific materials, systems and methods

We require information from authors about some types of materials, experimental systems and methods used in many studies. Here, indicate whether each material, system or method listed is relevant to your study. If you are not sure if a list item applies to your research, read the appropriate section before selecting a response.

Materials & experimental systems

n/a	Involved in the study
☐	☒ Antibodies
☐	☒ Eukaryotic cell lines
☒	☐ Palaeontology and archaeology
☒	☐ Animals and other organisms
☒	☐ Clinical data
☒	☐ Dual use research of concern
☒	☐ Plants

Methods

n/a	Involved in the study
☒	☐ ChIP-seq
☒	☐ Flow cytometry
☒	☐ MRI-based neuroimaging

Antibodies

Antibodies used	Anti-RPB1 antibody (BioLegend, cat. no. 920102, Clone 8WG16, Lot B217159, 500 uL antibody for 1 prep of Pol II from 114 L HeLa cells) was used for purification of endogenous Pol II.
Validation	Antibody specificity and performance was validated in this study by testing the purity of the purified Pol II by SDS-PAGE, verifying purified Pol II by negative-stain electron microscopy, and ultimately visualizing high-resolution structures by cryo-EM. The final structures confirm that the purified Pol II contains all subunits.

Eukaryotic cell lines

Policy information about cell lines and Sex and Gender in Research

Cell line source(s)	HeLa cells were obtained from the UC Berkeley Cell Culture Facility, and were used only for purification of endogenous human Pol II and not for any functional experiments.
Authentication	HeLa cells were authenticated by the UC Berkeley Cell Culture Facility using a GenePrint 10 kit (Promega) for short tandem repeat processing.
Mycoplasma contamination	HeLa cells were tested for mycoplasma by the UC Berkeley Cell Culture Facility and were negative.
Commonly misidentified lines (See ICLAC register)	No commonly misidentified cell lines were used in this study.

Plants

Seed stocks	N/A
Novel plant genotypes	N/A
Authentication	N/A
