# Supplementary material for: Structural insights into transcriptional regulation by the helicase RECQL5
Source: Nat Struct Mol Biol. 2025 Jul 7;32(9):1721–30. doi: 10.1038/s41594-025-01611-8 (PMC12262184; doi:10.1038/s41594-025-01611-8)
Supplement: Supplementary file 4 — Uncropped gel. [file 41594_2025_1611_MOESM4_ESM.pdf]

Source Data for Fig. 4h

Raw image

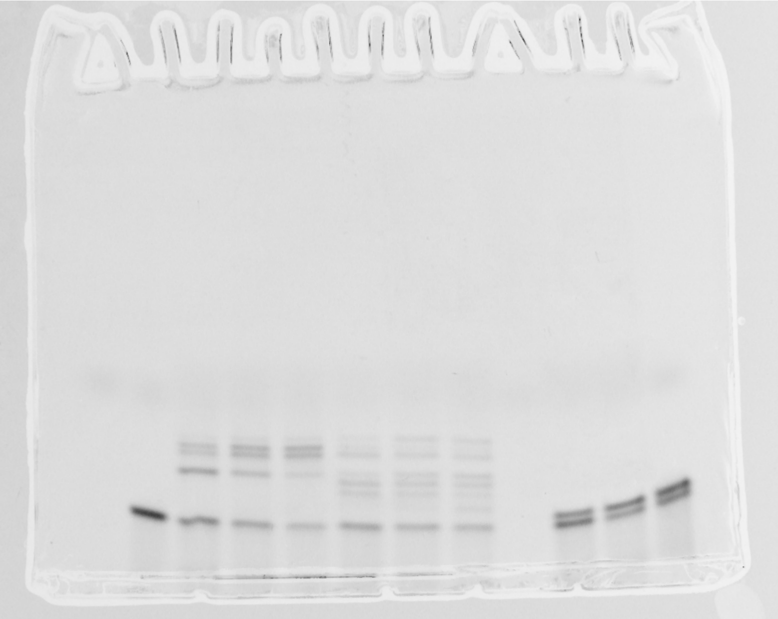

Cy3 fluorescence

Uncropped image (after tonal range adjustment)

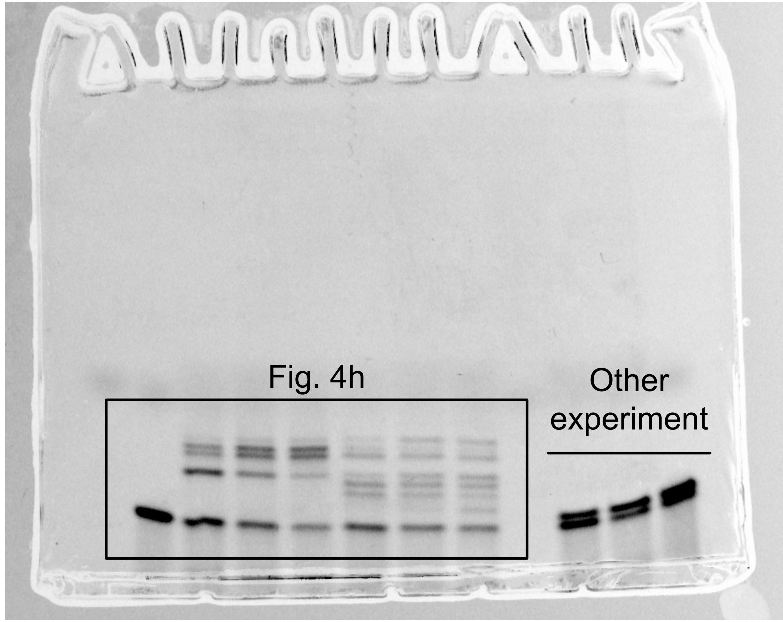

Cy3 fluorescence

Subsequent Sybr Gold staining to visualize ladder

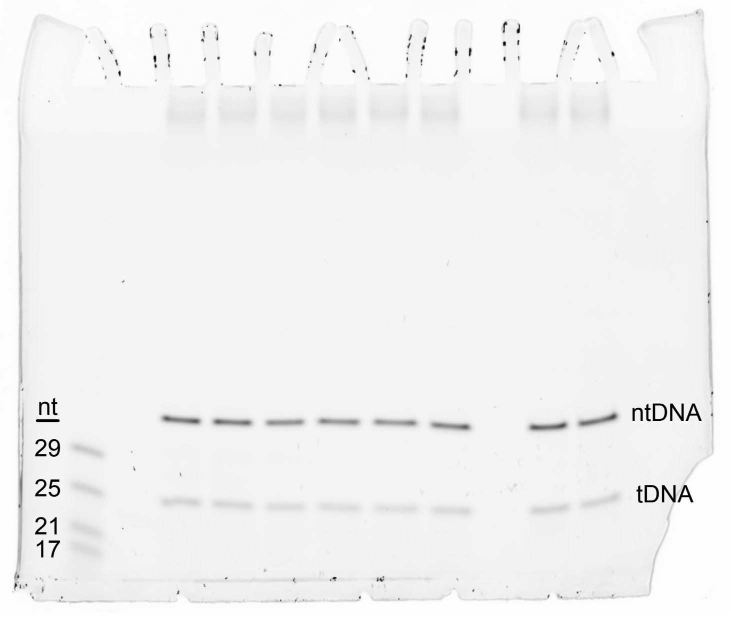

Sybr Gold fluorescence
