# Supplementary figures and images for: Structural insights into transcriptional regulation by the helicase RECQL5
Source: Nat Struct Mol Biol. 2025 Jul 7;32(9):1721–30. doi: 10.1038/s41594-025-01611-8 (PMC12262184; doi:10.1038/s41594-025-01611-8)

# Source Data for Extended Data Fig. 7b

Uncropped gels showing purified proteins/complexes

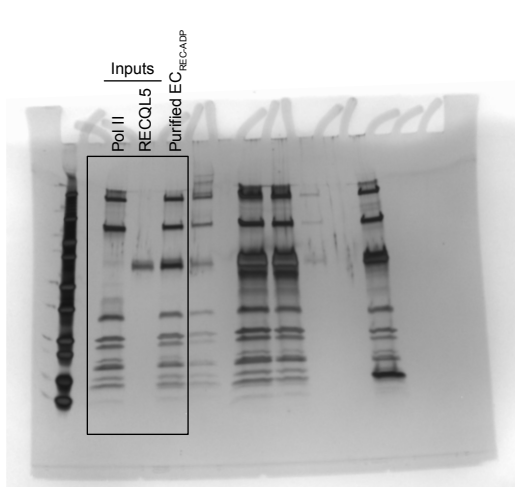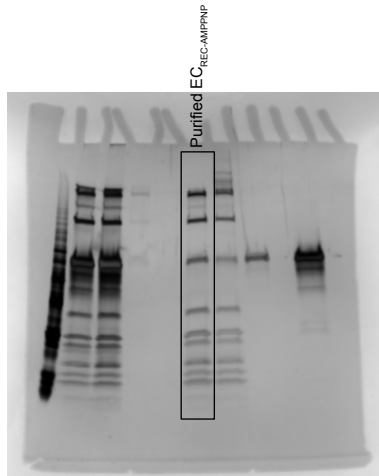

Supplement: Supplementary file 5 — Uncropped gels. [file 41594_2025_1611_MOESM5_ESM.pdf]
