# Supplementary material for: Structural insights into transcriptional regulation by the helicase RECQL5
Source: Nat Struct Mol Biol. Author manuscript; Available in PMC 2025 Jul 15. (PMC12262184; doi:10.1038/s41594-025-01611-8)
Supplement: Supplementary file 1 [file NIHMS2094604-supplement-Supplementary_Figure_1.pdf]

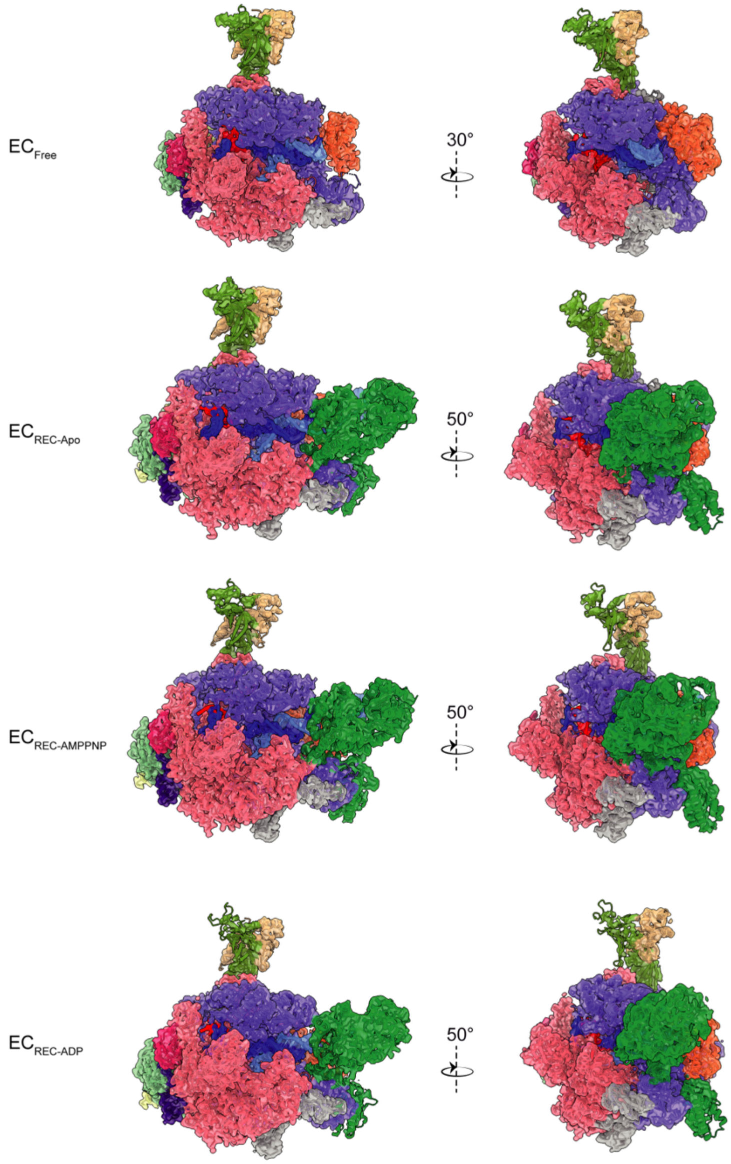

Supplementary Fig. 1 | Additional views showing model fit to map. Cryo-EM maps (shown in transparency) with fitted atomic models (shown in ribbon representation). Each map/model pair is shown in two distinct views.
